# Mammalian Target of Rapamycin Is a Therapeutic Target for Murine Ovarian Endometrioid Adenocarcinomas with Dysregulated Wnt/β-Catenin and PTEN

**DOI:** 10.1371/journal.pone.0020715

**Published:** 2011-06-09

**Authors:** Pradeep S. Tanwar, LiHua Zhang, Tomoko Kaneko-Tarui, Michael D. Curley, Makoto M. Taketo, Poonam Rani, Drucilla J. Roberts, Jose M. Teixeira

**Affiliations:** 1 Vincent Center for Reproductive Biology, Department of Obstetrics, Gynecology, and Reproductive Biology, Massachusetts General Hospital and Harvard Medical School, Boston, Massachusetts, United States of America; 2 Department of Pharmacology, Graduate School of Medicine, Kyoto University, Kyoto, Japan; 3 Department of Pathology, Massachusetts General Hospital and Harvard Medical School, Boston, Massachusetts, United States of America; Florida International University, United States of America

## Abstract

Despite the fact that epithelial ovarian cancers are the leading cause of death from gynecological cancer, very little is known about the pathophysiology of the disease. Mutations in the WNT and PI3K pathways are frequently observed in the human ovarian endometrioid adenocarcinomas (OEAs). However, the role of WNT/β-catenin and PTEN/AKT signaling in the etiology and/or progression of this disease is currently unclear. In this report we show that mice with a gain-of-function mutation in β-catenin that leads to dysregulated nuclear accumulation of β-catenin expression in the ovarian surface epithelium (OSE) cells develop indolent, undifferentiated tumors with both mesenchymal and epithelial characteristics. Combining dysregulated β-catenin with homozygous deletion of PTEN in the OSE resulted in development of significantly more aggressive tumors, which was correlated with inhibition of p53 expression and cellular senescence. Induced expression of both mTOR kinase, a master regulator of proliferation, and phosphorylation of its downstream target, S6Kinase was also observed in both the indolent and aggressive mouse tumors, as well as in human OEA with nuclear β-catenin accumulation. Ectopic allotransplants of the mouse ovarian tumor cells with a gain-of-function mutation in β-catenin and PTEN deletion developed into tumors with OEA histology, the growth of which were significantly inhibited by oral rapamycin treatment. These studies demonstrate that rapamycin might be an effective therapeutic for human ovarian endometrioid patients with dysregulated Wnt/β-catenin and Pten/PI3K signaling.

## Introduction

Ovarian cancer is the most deadly gynecological cancer among women in the United States with approximately 22,000 new cases diagnosed and 15,000 deaths yearly, the vast majority of which are from metastatic epithelial-derived ovarian tumors. The prognosis is poor because most patients are diagnosed during the late stage of the disease, when ovarian cancer has already metastasized and the survival rate is less than 30% [1], [2]. The cell of origin and course of disease progression is not well defined because cancer is usually diagnosed at an advanced stage [3]. The prevailing theory is that these tumors originate in the ovarian surface epithelium (OSE), a single layer of mesothelial cells covering the surface of the ovary [4], by a mechanism that might involve the formation of cortical inclusion cysts during rupture and repair after ovulation or from ovarian atrophy with aging [3].

Ovarian epithelial tumors are classified into five different major histological categories: serous, endometrioid, mucinous, clear cell, and undifferentiated [5]. It is not known whether a specific combination of genetic mutations force the OSE cells to acquire the specific lineage or whether tumors arise first and acquire more mutations leading to or commensurate with their commitment to Müllerian metaplasia later during the course of disease development. The latter hypothesis is well supported by studies in various mouse models of ovarian cancer in which initial OSE derived cancerous growths are undifferentiated [2], [6], [7].

Wnt signaling is essential for normal ovarian development and various members of the canonical Wnt signaling pathway are expressed in the ovary [8], [9]. Mutations in the β-catenin, APC, Axin1, and Axin2 genes are associated with ovarian epithelial cancers [10], [11]. Dysregulated WNT/β-catenin signaling plays an important role in the development of human ovarian endometrioid adenocarcinomas (OEAs) but is rare in other types of ovarian cancer. For example, mutations in exon 3 of β-catenin, which lead to its stabilization and nuclear accumulation [12], are present in an estimated 16%–38% of human OEAs [11], [13]. In the present report, we have investigated the mechanisms of tumorigenesis in murine OSE cells with deletion of exon 3 of β-catenin and/or Pten. We show that the mTOR pathway is activated in mice with dysregulated WNT/β-catenin and Pten/PI3K signaling, as well as in human OEAs. We also show evidence that rapamycin decreases tumor burden in allotransplants of the tumor cells, suggesting that human OEAs might be a good target for rapamycin therapy.

## Materials and Methods

### Mouse genetics and husbandry

All protocols involving animal experimentation were approved by the MGH Institutional Animal Care and Use Committee (Protocol# 2005N000195). The mice used in this study were maintained on C57BL/6;129/SvEv mixed genetic background and housed under pathogen free standard animal housing conditions as described [14]. The following parental mice alleles—*Ctnnb1^tm1Mmt^* [12], *Pten^tm1Hwu^* [15], *Amhr2^tm3(cre)Bhr^* [16]—were used in the crosses and are hereafter called *Ctnnb1^fl(ex3)^ or Ctnnb1^Δ(ex3)^*, *Pten^fl^*, or *Pten^Δ/Δ^*, *and Amhr2-Cre*, respectively. The genotyping of mice was performed with standard PCR protocols using DNA collected from tail biopsies. The PCR conditions for *Ctnnb1^fl(ex3)^* and *Amhr2^tm3(cre)Bhr^* are previously described [12], [17], [18]. The *Pten^fl^* allele was detected with primers 5′-ACTCAAGGCAGGGATGAGC-3′ and 5′-GCCCCGATGCAATAAATATG-3′, for 35 cycles of 94 C for 30 sec, 60 C for 1 min, and 72 C for 1 min using Taq DNA polymerase (Roche). The gross images were taken with a Nikon D60 digital camera with a macro lens.

### Histology and Immunofluorescence

The methods used for histology, IF, and IHC have been previously described [19]. IF and/or IHC was performed on tissues derived from minimum of three different animals per genotype. The whole tissue sections and/or at least three independent random areas of section at 10× were examined for specific markers staining and representative images are presented. The tissue blocks for human ovarian endometrioid adenocarcinomas (n = 4) and normal/benign ovarian samples (n = 3) were obtained from the Department of Pathology, MGH using Institutional Review Board-approved protocols. To examine epithelial glands, whole mouse ovarian tumors (n = 6) were cut into multiple pieces. The tumors blocks were serially sectioned and every 4^th^ section was stained for pancytokeratin or cytokeratin 8. The primary and secondary antibodies used in this study are described in Table 1. AlexaFluor secondary antibodies (1∶500, Invitrogen, Carlsbad, CA), biotinylated donkey anti-mouse or anti-rabbit antibody F_ab2_ (1∶1000, Jackson ImmunoResearch Laboratories, West Grove, PA), and DAB kit (Vector laboratories, Burlingame, CA) were used in this study. For senescence analysis, normal and mutant ovaries were collected and fixed overnight in 4% paraformaldehyde. The following day, frozen sections were cut and SAβ-gal staining was performed as instructed by the manufacturer (Cell Signaling Technology, Danvers, MA). Images were taken with a Nikon T2000 microscope equipped with an epifluorescence attachment and a Spot digital camera (Diagnostic Instruments, Sterling Heights, MI).

**Table 1:** Antibodies used in this study for IHC, IF and Western blot (WB) analyses.

ANTIGEN	SUPPLIER
p19^ARF^ , p21	Abcam, Cambridge, MA
Activated Caspase 3, β-catenin	BD Transduction Laboratories, San Jose, CA
Vimentin, AMH, p16^INK4A^	Santa Cruz Biotechnology, Santa Cruz, CA
Inhibin-α	Biogenes, San Ramon, CA
pH3, γ-H2AX	Millipore, Bellerica, MA
α-SMA-Cy3, β-catenin	Sigma Chemical Co., St. Louis, MO
AKT, phosphoAKT, Pten, p53, mTOR, pmTOR, S6K, pS6K, and p27kip1	Cell Signaling Technologies, Danvers, MA
Pan-cytokeratin	Neomarkers, Fremont, CA
Cytokeratin 8	Developmental studies hybridoma bank, IA
β-actin	Neomarkers, Fremont, CA

### Primary tumor cell isolation, transplantation to NOD/SCID mice, and rapamycin treatments

Primary ovarian tumors were derived from the *Amhr2-Cre;Ctnnb1^Δ(ex3)/+^;Pten^Δ/Δ^* mice. Cells from the primary tumors were isolated, diluted (2×10^6^ cells/mouse) in 1∶1 Matrigel (BD Biosciences), and injected subcutaneously into female NOD/SCID mice (The Jackson Laboratory, Bar Harbor, ME). Grafted mice were divided into two groups (n = 5/group) and administered 200 µl rapamycin (250 µg/dose, Rapamune, Wyeth, PA) or vehicle control (kindly donated by The American Lecithin Company, Oxford, CT) twice/week by oral gavage. After 10 weeks of treatment, tumors were excised for analysis. All animals were euthanized after 12 weeks.

### Western blot analyses

Western analyses of ovarian cells collected from a minimum of 3 age-matched control and mutant mice were performed as previously described [20]. β-actin was used as a loading control. The experiments were repeated three times. Granulosa cells were isolated from mice 24 h after they were injected with pregnant mare's serum gonadotropin by puncturing the follicles with a tuberculin syringe.

### Tumor Morphometrics

At the end of study, tumor volume was calculated based on caliper measurements of the exposed tumor using the formula for an ellipsoid: 4/3 π×1/2 length×(1/2 width)^2^ [21]. Terminal deoxynucleotidyl Transferase Biotin-dUTP Nick End Labeling (TUNEL) staining was performed per the manufacturer's instructions (Roche, Indianapolis, IN). For estimation of the total number of pH3-, active caspase 3- and TUNEL-positive cells, images from three different tumors derived from three different animals were taken at equivalent gain settings using a microscope (Nikon TE 2000-S; Micro Video Instruments, Avon, MA) equipped with a Spot digital camera. pH3-, active caspase 3-, and TUNEL-positive cells, and DAPI stained nuclei were counted with the nucleus-counting plugin of Image J software (v1.37, NIH, Bethesda, MD) after setting a fixed threshold in Photoshop (v10; Adobe systems Inc., San Jose, CA).

### Statistical analysis

The unpaired *t* test was used to test for differences between groups and p<0.05 was considered statistically significant. Statistical analyses were performed using Prism software (GraphPad Software, San Diego, CA).

## Results

### Deregulated Wnt/β-catenin signaling in human and mouse ovarian tumors

**Figure 1:**
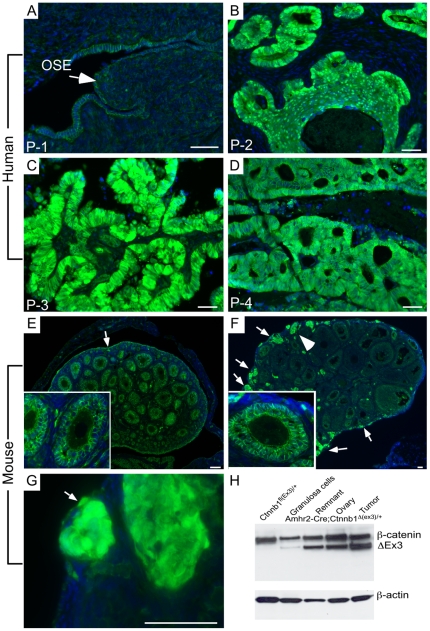
Overactive Wnt/β-catenin in human OEAs and mutant *Amhr2-Cre;Ctnnb1^Δ(ex3)/+^* mice. (A) Membranous β-catenin expression in normal postmenopausal human ovarian surface epithelium (OSE) was detected by immunofluorescence. (B–D) Nuclear β-catenin in representative tissue samples obtained from three different human OEA patients. β-catenin protein expression in control *Ctnnb1^Δ(ex3)/+^* (E) and in *Amhr2-Cre;Ctnnb1^Δ(ex3)/+^* (F) ovaries of 4-week-old mice. Insert in Panels E and F are higher magnification images of follicles to show membranous β-catenin expression in granulosa cells. (G) Higher magnification image of area indicated with a white arrowhead in panel F. (H) Western blot analyses of β-catenin in protein lysate obtained from granulosa cells, remnant (remaining ovarian tissue after granulosa cell isolation including ovarian surface epithelium), whole control (*Ctnnb1^Δ(ex3)/+^*) and mutant (*Amhr2-Cre;Ctnnb1^Δ(ex3)/+^*) 4-week-old ovaries, tumors from the mutant (*Amhr2-Cre;Ctnnb1^Δ(ex3)/+^*) 6-month-old ovaries. White arrows in A, E, F, and G mark the ovarian surface epithelial cells. Nuclei are stained with DAPI. Bars = 50 um.

Because mutations in Wnt/β-catenin signaling components are frequently observed in human OEAs patients [11], [13], we examined β-catenin protein expression in human OEA tissue samples and observed nuclear accumulation of β-catenin, which is indicative of Wnt pathway activation, in 80% (4/5) (Fig. 1B–D). Only membranous β-catenin protein expression was observed in OSE of normal human ovary (Fig. 1A). To confirm that activated Wnt/β-catenin signaling can initiate the formation of OEAs, we developed a β-catenin gain-of-function mouse model by conditionally deleting exon 3 (Ex3) of the β-catenin gene (*Ctnnb1*), which contains the phosphorylation sites of β-catenin required for its ubiquitination and subsequent degradation [12]. Deletion of exon 3 leads to formation of a degradation-resistant but functional form of β-catenin, which accumulates in the cytoplasm and nuclei of cells [12]. Amhr2-cre mice were used to mate with mice with a flox allele of β-catenin, *Ctnnb1^fl(ex3)/+^* because Amhr2 is expressed in OSE and *Amhr2* promoter-driven Cre causes efficient recombination in OSE cells [2], [19], [22], [23], [24]. In order to verify whether activation of β-catenin occurs in OSE cells of *Amhr2-Cre;Ctnnb1^Δ(ex3)/+^* ovaries, we analyzed expression of β-catenin. Examination of four-week-old mice showed nuclear accumulation of β-catenin in OSE and OSE-derived lesions present throughout the cortex of the mutant *Amhr2-Cre;Ctnnb1^Δ(ex3)/+^* ovaries (Fig. 1F & G)). In contrast, only membranous β-catenin expression was observed in the OSE of age matched control ovaries (Fig. 1E). Membranous β-catenin expression was also observed in the granulosa cells of both mutant and control *Ctnnb1^fl(ex3)/+^* ovaries (Fig. 1E & F, inserts). We next performed western blot analyses on ovarian lysates and demonstrated the presence of full length and exon 3-deleted β-catenin in the granulosa cell-depleted residual ovarian cells, including those from OSE and tumor in the mutant animals (Fig. 1H). A relatively weaker band of exon 3-deleted β-catenin was detected in the granulosa cells of the mutant ovaries indicating that very low recombination occurred in granulosa cells, which is consistent with the largely membranous staining observed in Fig. 1F or that the granulosa cell preparations were contaminated with ovarian stromal cells.

**Figure 2:**
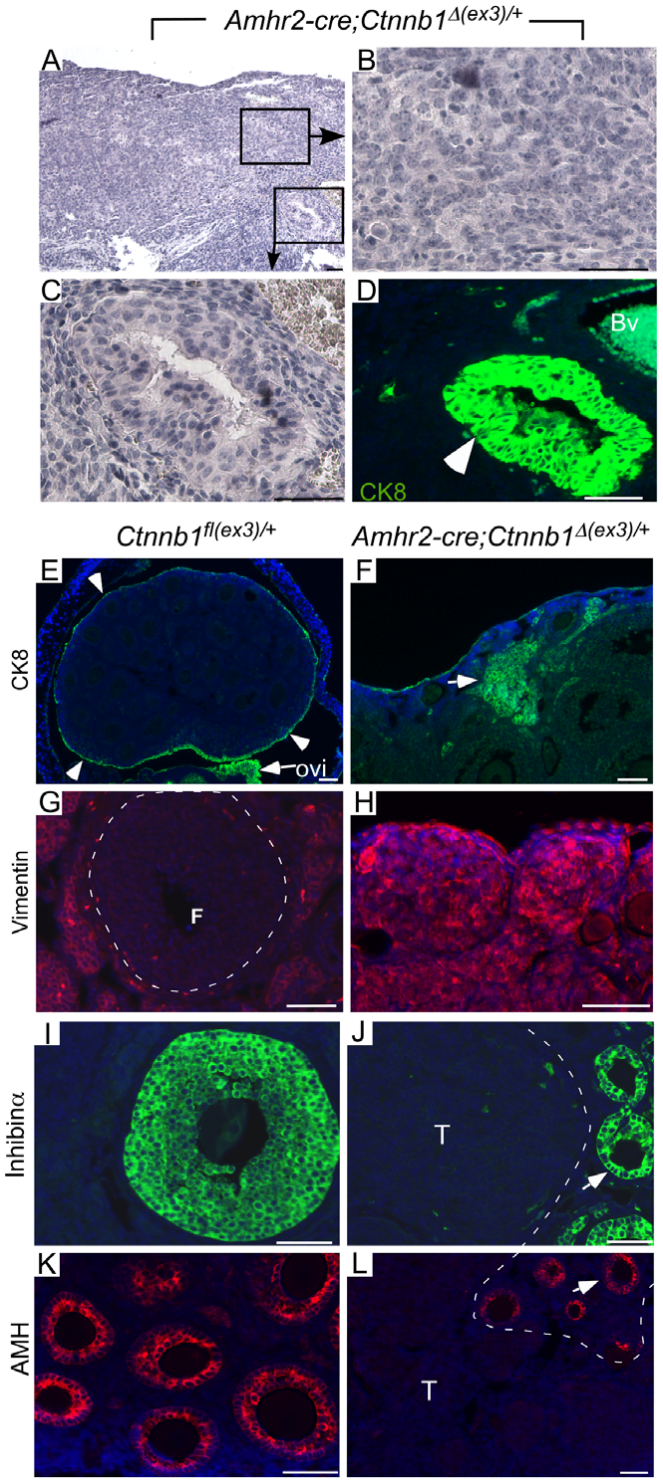
Histological examination of ovarian tumors formed in *Amhr2-Cre;Ctnnb1^Δ(ex3)/+^* mice ovaries. H&E staining of mutant ovaries (Panels A–C). Panels B & C are magnified views of boxed areas in Panel A. Cytokeratin 8 (CK8) immunofluorescence on a serial section (D) of panel C confirming presence of epithelial glands (arrowhead); Bv indicates background fluorescence from a blood vessel. (Panel E) CK8 staining in control ovary marks OSE cells (arrowheads) and oviductal (Ovi) epithelial cells. (Panel F) CK8 expression in a typical small tumor in *Amhr2-Cre;Ctnnb1^Δ(ex3)/+^* ovaries. (Panel G) Vimentin immunofluorescence in control ovaries was present in some stromal cells but not in granulosa cells of follicles (outlined by dotted line, F). In *Amhr2-Cre;Ctnnb1^Δ(ex3)/+^* ovaries (Panel H), vimentin expression was observed throughout the tumors. (Panels I–L) Amh and inhibin-α expression in control (I & K) and mutant (J & L) ovaries. Inhibin-α and Amh expression was present in granulosa cells of the remnant follicles (arrow, demarcated from the tumor by white dotted line) of mutant ovaries but not in tumor areas (indicated with a T). Nuclei are stained with DAPI in Panels D–L. Bars = 50 um.

Histological examination of adult mutant ovaries (≥12-wks of age) revealed the presence of pre-tumoral nests of cells in all the ovaries examined in this study (n = 10) (Fig. S1C–E). The cancerous cells were also present between the ovarian bursa and OSE (Fig. S1C–E). Nuclear β-catenin was observed in these pre-tumoral nests, indicating that these lesions were derived from Amhr2-cre expressing cells (Fig. S1F). Ovaries from age-matched control mice appeared morphologically normal (Fig. S1A & B). Even though pretumoral lesions were present in the ovaries of all young *Amhr2-Cre;Ctnnb1^Δ(ex3)/+^* mice, advanced tumor development only occurred in approximately 50% of mice by the age of 8-month to 1-year (Fig. 2). While the majority of the tumors were undifferentiated (Fig. 2A & B), epithelial glands, which are characteristic of ovarian endometrioid adenocarcinomas, were observed in 5/6 mice examined (Fig. 2C). Immunostaining with cytokeratin 8 (CK8), an epithelial cell specific marker, confirmed the presence of epithelial glands in these tumors (Fig. 2D). CK8-specific staining was also observed in early pretumoral lesions, as well as in undifferentiated tumors, indicative of the epithelial cell origin of these tumors (Fig. 2F). In control ovaries, CK8 staining was only observed in the ovarian surface epithelium and oviductal epithelial cells (Fig. 2E). Because the less differentiated areas of *Amhr2-Cre;Ctnnb1^Δ(ex3)/+^* tumors showed weak staining for CK8 and had a more spindle shape morphology, we examined the expression of vimentin, a mesenchymal marker. We observed strong positive staining for vimentin in less differentiated areas of pretumoral lesions and in fully-grown tumors, suggesting that cancerous cells undergo epithelial mesenchymal transition (EMT) in these tumors (Fig. 2H). Vimentin staining was observed in stromal but not in granulosa cells of the control ovaries (Fig. 2G).

Since these tumors showed less differentiated morphology and Amhr2-cre is also expressed in granulosa cells at later stages of development [25], we examined expression of the ovarian granulosa cell tumor markers to rule out the possibility that these tumors might be derived from the granulosa cells. We analyzed the expression of inhibin-α and anti-Müllerian hormone (AMH, also known as Müllerian Inhibiting Substance [26]), which are the two markers most often used to detect murine, human, and equine granulosa cell tumors [27], [28], [29], in *Amhr2-Cre;Ctnnb1^Δ(ex3)/+^* tumors (Fig. 2I–L). Although expression of both was observed in control follicles (Fig. 2I & K) and in the remaining follicles of early tumors (Fig. 2J & L), expression of these two markers was not observed in the tumorous areas.

### PTEN deletion with constitutively activated (CA) β-catenin leads to development of more aggressive tumors

**Figure 3:**
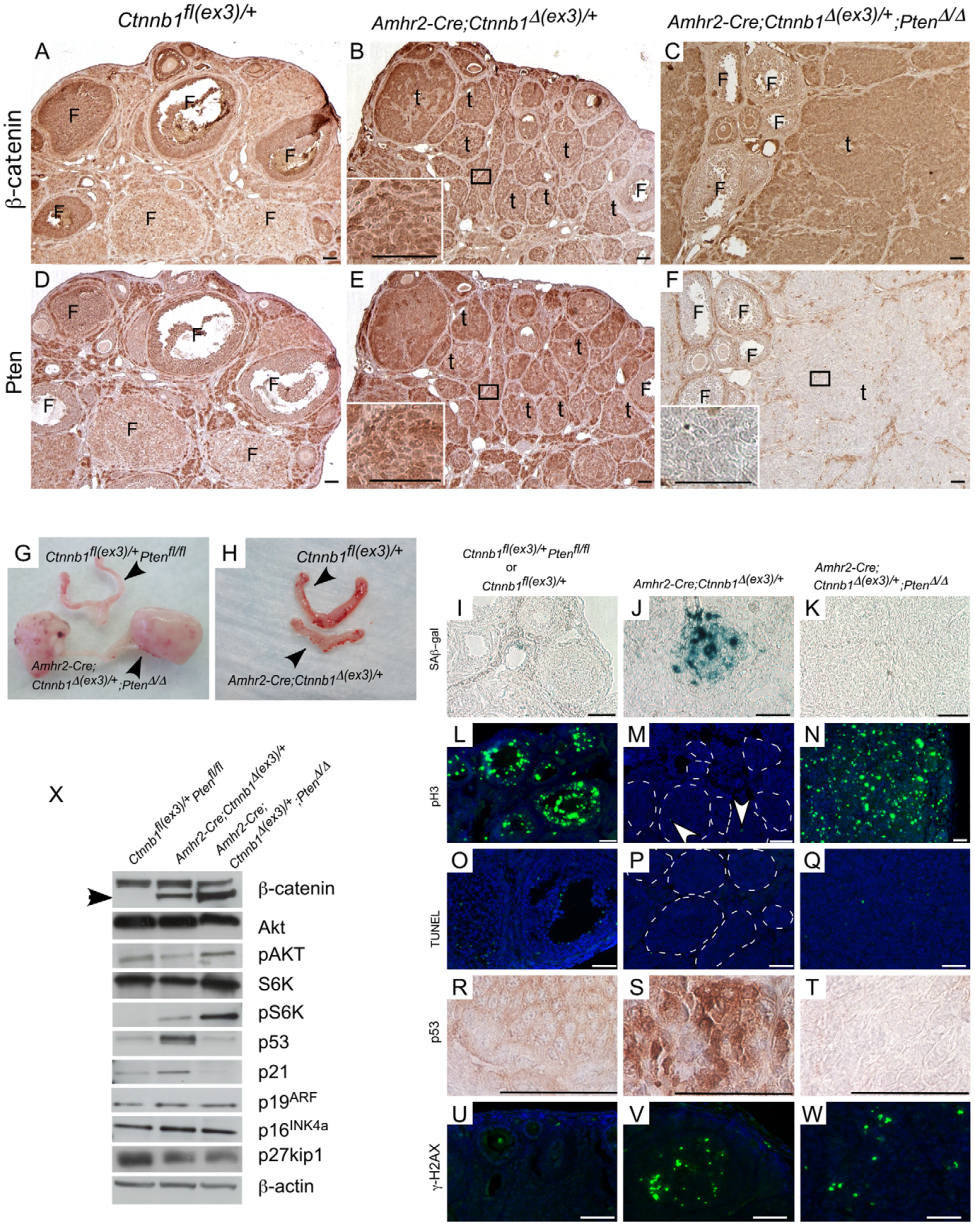
Sustained activation of β-catenin in the somatic cells leads to the induction of p53-mediated senescence in *Amhr2-Cre;Ctnnb1^Δ(ex3)/+^* ovaries. (Panel A–F) Colocalization of β-catenin and PTEN in serial sections of control (*Ctnnb1^Δ(ex3)/+^*), *Amhr2-Cre;Ctnnb1^Δ(ex3)/+^* and *Amhr2-Cre;Ctnnb1^Δ(ex3)/+^;Pten^Δ/Δ^* ovaries. Insets in Panels B, E, & F are higher magnification images of areas marked by black rectangles. F: follicle, t: tumor. (Panel G & H) Gross analyses of the reproductive tracts from the indicated mice show large tumors by 6 weeks in *Amhr2-Cre;Ctnnb1^Δ(ex3)/+^;Pten^Δ/Δ^* ovaries but relatively normal size ovaries in *Amhr2-Cre;Ctnnb1^Δ(ex3)/+^* mice . SAβ-gal staining was observed in *Amhr2-Cre;Ctnnb1^Δ(ex3)/+^* ovaries but not in control or *Amhr2-Cre;Ctnnb1^Δ(ex3)/+^;Pten^Δ/Δ^* ovaries (I–K). Compared to controls and *Amhr2-Cre;Ctnnb1^Δ(ex3)/+^;Pten^Δ/Δ^* ovaries, very few pH3 positive cells were present in precancerous lesions (white dotted lines) of *Amhr2-Cre;Ctnnb1^Δ(ex3)/+^* ovaries (L–N). Absence of TUNEL staining in pretumoral lesions (white dotted lines) showed that non-proliferating cells in *Amhr2-Cre;Ctnnb1^Δ(ex3)/+^* ovaries are not apoptotic (O–Q). Immunohistochemical analyses showed an increase in p53 accumulation in *Amhr2-Cre;Ctnnb1^Δ(ex3)/+^* tumors compared to controls and loss of p53 expression in *Amhr2-Cre;Ctnnb1^Δ(ex3)/+^;Pten^Δ/Δ^* tumors (R–T). Evidence of DNA damage determined by staining for γ-H2AX and observed in the tumors of both mutants (V & W) but not in the control ovaries (U). The differential expression of p53 was confirmed by Western blot analyses with pooled ovarian lysates (n = 4) and repeated three times (Panel X), which also confirmed the presence of exon 3 deleted β-catenin (arrowhead) in *Amhr2-Cre;Ctnnb1^Δ(ex3)/+^;Pten^Δ/Δ^* ovaries. Analyses also showed increased expression of pAKT and pS6K, and decreased expression of p21 and p27kip1 in *Amhr2-Cre;Ctnnb1^Δ(ex3)/+^;Pten^Δ/Δ^* ovaries. β-actin was used as a loading control. Bars = 50 um.

Interactions between Wnt/β-catenin and Akt/PTEN signaling pathways play an important role in carcinogenesis [6]. We examined the status of PTEN by performing immunohistochemical staining for β-catenin and PTEN on serial sections of *Amhr2-Cre;Ctnnb1^Δ(ex3)/+^* ovaries and observed increased expression of PTEN in pretumoral lesions with nuclear β-catenin (Fig. 3B & E). To examine whether PTEN deletion could affect tumor progression in *Amhr2-Cre;Ctnnb1^Δ(ex3)/+^* ovaries, we developed another mouse model by deleting exon 3 of the β-catenin in PTEN negative cells of the mouse ovary (*Amhr2-Cre;Ctnnb1^Δ(ex3)/+^;Pten^Δ/Δ^*) (Fig. 3C & F). These mice showed early onset of tumor development and were euthanized because of tumor-related morbidities (Fig. 3G & H). No evidence of ascites or metastases was observed by gross examination of the peritoneal cavity. The ovarian tumors in *Amhr2-Cre;Ctnnb1^Δ(ex3)/+^;Pten^Δ/Δ^* ovaries showed histopathological features similar to the tumors of *Amhr2-Cre;Ctnnb1^Δ(ex3)/+^* mice (Fig. S2). Consistent with previous observations [22], [30], deletion of PTEN alone does not result in ovarian tumor development (data not shown), suggesting that activation of the phosphatidylinositol-3′ kinase (PI3K) signaling pathway with inactivating PTEN mutations alone may not be sufficient for tumor initiation and needs to act in concert with other oncogenes to cause cancer.

PTEN has been shown to regulate P53 protein levels and this interaction plays a role in cellular senescence, growth inhibition, and cellular transformation [31]. Additionally, dysregulated β-catenin expression causes growth arrest and a senescent-like state in mouse embryonic fibroblast cells (MEFs) and activation of β-catenin stimulates proliferation of p53-deficient MEFs suggesting that the senescence observed after activation of β-catenin is mediated by p53 [32]. In vivo, p53 deficiency increases the tumorigenicity of intestinal tumors in APC-deficient mice indicating that loss of p53 plays an important role in the progression of these tumors [33]. We have observed early onset of tumor development in *Amhr2-Cre;Ctnnb1^Δ(ex3)/+^;Pten^Δ/Δ^* mice (Fig. 3G & H). However, tumor progression has up to a year long latency period in *Amhr2-Cre;Ctnnb1^Δ(ex3)/+^* mice (Fig. 3H) and no change was observed in the mortality rate of these animals, even though the pretumoral lesions were present in 4-week old ovaries (Fig. 1). We examined whether the p53 senescence pathway might be involved in the inhibition of tumor progression in the *Amhr2-Cre;Ctnnb1^Δ(ex3)/+^* ovaries, which could account for the discrepancy in timing of tumor progression in these two genotypes. We found positive staining for SAβ-gal, a hallmark of senescent cells [34], in pretumoral lesions present in the *Amhr2-Cre;Ctnnb1^Δ(ex3)/+^* ovaries (Fig. 3J). In contrast, SAβ-gal staining was absent in the *Amhr2-Cre;Ctnnb1^Δ(ex3)/+^;Pten^Δ/Δ^* and control ovaries (Fig. 3I & K). We then examined the proliferation index of these tumors by phospho-histone H3 (pH3) immunofluorescence, a marker for mitotic cells [35]. Very few pH3-positive cells were present in the pretumoral lesions of *Amhr2-Cre;Ctnnb1^Δ(ex3)/+^* ovaries (Fig. 3M). However, many more pH3-positive cells were present in the *Amhr2-Cre;Ctnnb1^Δ(ex3)/+^;Pten^Δ/Δ^* tumors and control ovaries (Fig. 3L & N). To ensure that these non-proliferating cells in the pretumoral ovaries are not dead cells, we performed a TUNEL assay. The cells present in the indolent pretumoral lesions of the *Amhr2-Cre;Ctnnb1^Δ(ex3)/+^* ovaries were TUNEL-negative (Fig. 3P) and only a few TUNEL-positive cells were present in *Amhr2-Cre;Ctnnb1^Δ(ex3)/+^;Pten^Δ/Δ^* tumors (Fig. 3Q). As an endogenous positive control for the assay, some TUNEL positive cells were present in the atretic follicles of the control ovaries (Fig. 3O).

To examine the possible mechanisms involved in the induction of senescence in the *Amhr2-Cre;Ctnnb1^Δ(ex3)/+^* ovaries, we performed western blot and IHC analyses of various proteins involved in the induction of senescence [36]. We found increased expression of p53 (Fig. 3S & X) and p21 (Fig. 3X) in the *Amhr2-Cre;Ctnnb1^Δ(ex3)/+^* ovaries. However, p53 and p21 expression were decreased in ovaries also deleted for PTEN (Fig. 3T & X), suggesting that the p53-p21 senescence pathway was functional and responsible for tumor inhibition in *Amhr2-Cre;Ctnnb1^Δ(ex3)/+^* ovaries. We also investigated whether the elevated levels of p53 and p21 might be the related to increased DNA damage, an early event in tumorigenesis and showed that γ-H2AX, the phosphorylated form of the core histone H2AX and a marker for double-stranded DNA breakage, was highly expressed in both *Amhr2-Cre;Ctnnb1^Δ(ex3)/+^* and *Amhr2-Cre;Ctnnb1^Δ(ex3)/+^;Pten^Δ/Δ^* tumor cells compared to control ovarian tissue (Fig. 3 U–W). Unlike most in vitro studies [32], we observed no noticeable difference in p19^arf^ and p16^ink4a^ protein levels suggesting limited involvement of these cell cycle regulators in these in vivo model systems (Fig. 3X). The expression of another cell cycle regulator, p27^kip1^, was marginally reduced in both *Amhr2-Cre;Ctnnb1^Δ(ex3)/+^* and *Amhr2-Cre;Ctnnb1^Δ(ex3)/+^;Pten^Δ/Δ^* tumors compared to control (Fig. 3X). Additionally, protein levels of phospho-S6 kinase (pS6K) were increased in *Amhr2-Cre;Ctnnb1^Δ(ex3)/+^* and *Amhr2-Cre;Ctnnb1^Δ(ex3)/+^;Pten^Δ/Δ^* ovaries, suggesting that the mTOR pathway is activated during development of these tumors (Fig. 3X).

### CA β-catenin induces mTOR expression and rapamycin treatment reduces tumor burden

**Figure 4:**
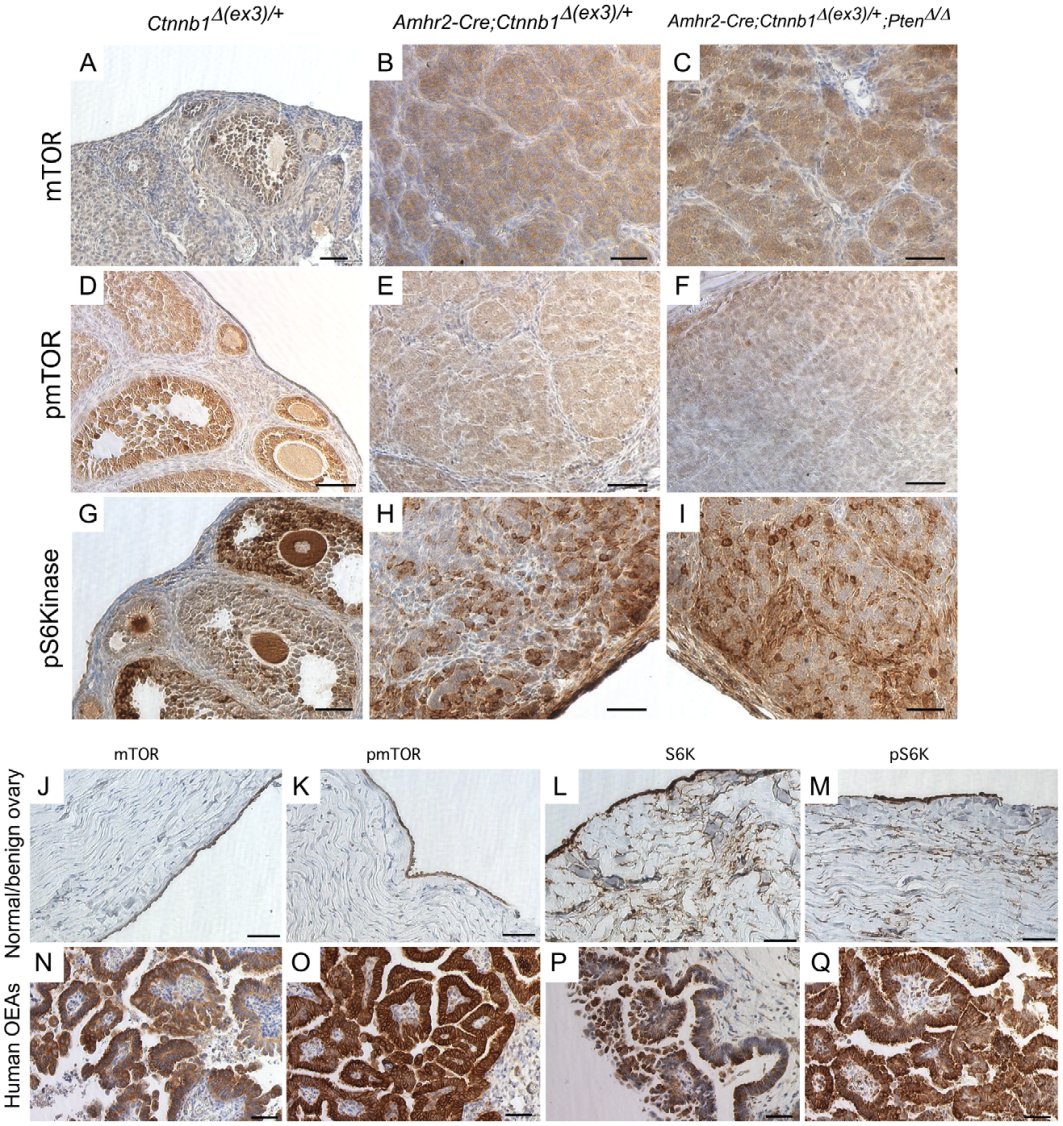
Elevated levels of active mTOR in mouse ovarian tumors and in human OEAs. Sections from ovaries of control (A, D, & G) and mutant mice (B, C, E, F, H, & I) were analyzed by IHC for mTOR, pmTOR, and pS6K, as indicated. IHC of mTOR (J & N) , pmTOR (K & O), S6K (L & P) and pS6K (M & Q) in normal postmenopausal ovaries (n = 3) and in human OEAs (n = 4) as indicated. Bars = 50 um.

Mammalian target of rapamycin (mTOR) signaling is a master regulator of cellular proliferation and is an attractive therapeutic target for a variety of cancers [37]. Dysregulated Wnt/β-catenin signaling has been shown to induce mTOR expression and signaling in intestinal polyps [38] of mutant APC mice, and we have shown that mTOR expression is elevated in uteri of *Amhr2-Cre;Ctnnb1^Δ(ex3)/+^* mice [19]. In Fig. 3X, we observed elevated expression by western analysis of pS6K, a downstream target of mTOR kinase activity, in the *Amhr2-Cre;Ctnnb1^Δ(ex3)/+^* and *Amhr2-Cre;Ctnnb1^Δ(ex3)/+^;Pten^Δ/Δ^* tumors compared to control mice. Immunohistochemistry of mTOR, p mTOR, and pS6K expression was performed to confirm that the increased expression was isolated to the tumor cells in mutant ovaries (Fig. 4A–I). We also observed strong expression for mTOR, p mTOR, and pS6K in human OEAs (4/4) (Fig. 4J–Q), which in Fig. 1, we showed also had induced levels of nuclear β-catenin.

**Figure 5:**
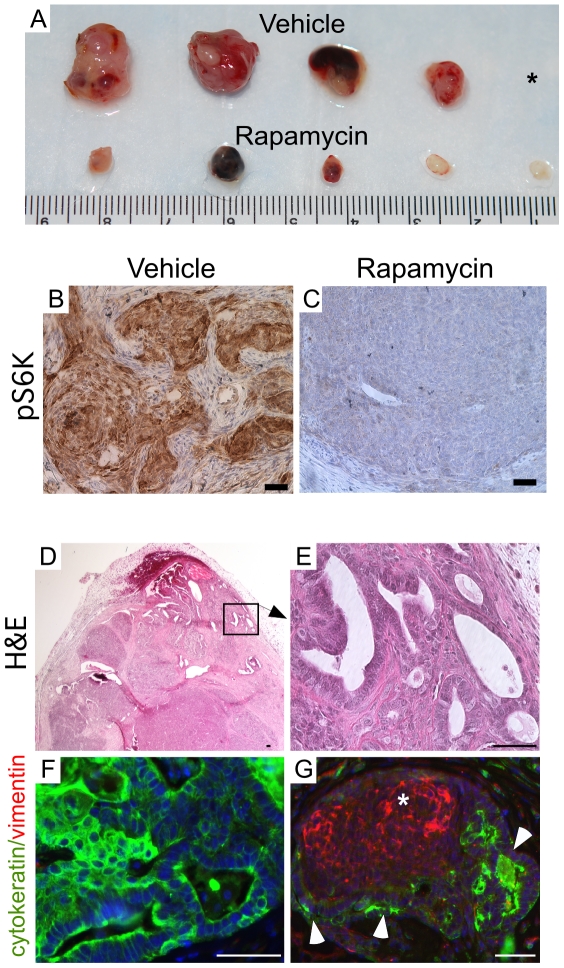
Rapamycin treatment reduced tumor burden by inhibiting mTOR activity and controlling cell proliferation and death. (Panel A) Tumor size was significantly decreased in rapamycin-treated mice compared to vehicle-treated controls. *One of the vehicle-treated mice was euthanized early because the tumor had grown too large and was excluded from morphometric measurements. (Panel B & C) pS6K immunostaining of tumors (n = 5) showed that vehicle-treated tumors were strongly positive; whereas rapamycin treated tumors (n = 5) were negative. (Panel D & E) H&E staining revealed distinct epithelial glands similar to primary tumors. Panel E is the higher magnification image of area marked by a rectangle in Panel D. Colocalization of vimentin (red) and cytokeratin (green) revealed that these tumors are strongly positive for cytokeratin (F). Occasional tumor lesions expressing both markers (G), vimentin (red, asterisk) and cytokeratin (green, arrowheads) were also observed. Bars = 50 um.

The upregulation of mTOR activity is commonly observed in colon, urinary bladder, and salivary gland cancers formed after dysregulated Wnt and/or PI3K signaling [38], [39], [40]. Since we observed increased pS6K protein levels in tumors formed in the mouse models (*Amhr2-Cre;Ctnnb1^Δ(ex3)/+^* and *Amhr2-Cre;Ctnnb1^Δ(ex3)/+^;Pten^Δ/Δ^*) and in human OEAs, we speculated that the mTOR pathway might also be involved in the growth of these tumors. Tumor cells were collected from *Amhr2-Cre;Ctnnb1^Δ(ex3)/+^;Pten^Δ/Δ^* mutant ovaries and injected into the dorsal flanks of 10 NOD/SCID mice in order control for uniformity at the start of the experiment. After the first week, five mice were randomly assigned to either a rapamycin-treated group or a vehicle-treated group. Significantly reduced tumor growth was observed in mice treated with rapamycin for 12 weeks by oral gavage, compared to the vehicle-treated controls (Fig. 5A). The tumor volume and weight were significantly lower in the rapamycin-treated group compared to the control group (Table 2). Rapamycin treatment in cancer cell lines and in vivo mouse models induces growth arrest and apoptosis [41]. We investigated the inhibitory effect of rapamycin in these tumors by analyzing the proliferation index and apoptotic rate compared to control-treated tumors (Fig. S3). Rapamycin-treated tumors had 1/3^rd^ the number of proliferating pH3-positive cells than the vehicle-treated tumors (Table 2), suggesting that rapamycin treatment decreased the proliferation rate of the tumor cells. TUNEL and activated Caspase 3 immunostaining showed that rapamycin treatment also resulted in a significant increase in cell death compared to the vehicle-treated tumors (Table 2 & Fig. S3). To confirm that rapamycin treatment inhibited the activation of mTOR signaling in the treated tumors, we performed immunohistochemical staining for pS6K. In Fig. 5B & C, we show a representative example from one of the 5 vehicle-treated tumors with strong pS6K immunostaining, whereas little pS6K immunostaining was detected in 100% (5/5) of the rapamycin-treated tumors examined. These findings indicate that rapamycin treatment significantly decreased tumor burden by inhibiting mTOR activity, mainly by reducing proliferation and increasing cell death. We also performed histological examination of vehicle- and rapamycin-treated tumors. Similar to primary tumors, grafted tumors showed epithelial glands admixed with less differentiated tumor cells (Fig. 5D & E). Colocalization of vimentin (a mesenchymal marker) and cytokeratin (an epithelial marker) in the grafted tumors showed that tumors were strongly positive for cytokeratin and some areas of tumors were positive for both markers (Fig. 5F & G), suggesting that epithelial to mesenchymal (EMT) transition might be occurring in the allotransplants.

**Table 2:** Morphometric Analyses of Tumor Grafts.

	Rapamycin;mean ± SEM	Vehicle;mean ± SEM	p value
Body weight (g)	22.2±0.3	22.5±0.7	0.97
Tumor volume (mm^3^)	5.9±3.7	702.2±256.9	0.02
Tumor weight (mg)	9.0±2.4	335.0±135.6	0.03
TUNEL^a^	62.0±12.1	6.3±2.0	0.01
pH3^a^	39.2±2.1	176.5±45.9	0.04
Activated Caspase 3^a^	10.2±1.8	1.4±0.1	0.01

a Positive cells/field.

## Discussion

Most ovarian carcinomas are thought to originate from the mesothelial cells covering of the ovary known as the OSE [4]. However, the histology of the most common human epithelial ovarian cancers is more similar to the fallopian tube (serous), uterus (endometrioid) and cervix (mucinous) [4]. It is not known how this simple monolayer of cells gives rise to such a complex disease and why ovarian carcinomas are so similar to the Müllerian duct-derived tissues. During development, Müllerian duct epithelia commit to the specific lineages by acquiring a fallopian, uterine, or cervical phenotypes due, in part, to the segmental expression of *Hoxa* genes [42], [43]. However, OSE cells maintain a primitive multipotential phenotype [44]. It has been suggested that over time these cells accumulate various genetic mutations leading to tumor formation and subsequent differentiation to the various lineages of ovarian tumors [4], [45]. Other studies have suggested alternative sites, like the distal end of fimbriae and secondary Müllerian duct system, as the points of origin for ovarian carcinomas [3], [45]. Additionally, endometriotic lesions have been suggested as the precursor for OEAs, particularly those with β-catenin and PTEN mutations [46]. Our results favor the OSE origin hypothesis, at least for OEA development, since we observed tumors arising in the OSE that can differentiate into the OEA histotype.

In human colorectal cancers, tumor cells with nuclear accumulation of β-catenin undergo growth arrest and EMT [47], [48]. These cells progressively lose E-cadherin, an epithelial marker, and acquire fibronectin expression, a mesenchymal marker [47], [48]. Furthermore, Wnt signaling has been shown to promote EMT and a tumor invasion of the breast and cervical cancer cells by regulating Snail activity [49]. The forced expression of Snail and Slug in ovarian cancer cell lines suppresses E-cadherin levels, which correlates with the induction of an EMT-like state and generation of cancer cells with stem cell characteristics [50]. In a previous report, it was observed that combined deletion of APC and PTEN causes formation of ovarian epithelial tumors accompanied by loss of E-cadherin expression and appearance of mesenchymal like-cells suggesting EMT [6]. Our results showing expression of both mesenchymal and epithelial markers in the tumors (Figs. 2 & 5) also suggested that EMT occurs during the development of ovarian carcinoma with dysregulated Wnt/β-catenin signaling. Preliminary studies to investigate this possibility showed upregulated expression of Snail and Slug in the tumors by immunofluorescence (data not shown) but more detailed mechanistic studies are required to determine whether dysregulated Wnt/β-catenin expression is driving EMT, which then leads to tumor development, or whether EMT is the result of tumor development.

In previous reports [51], [52], appearance of these mesenchymal cells was considered a malignant transformation of granulosa cells, which led to their classification as granulosa cell tumors of the ovary, even though the markers for granulosa cells were not reported. However, we showed that these tumors failed to express markers of granulosa cell tumors (AMH and inhibin-α), and express epithelial markers suggesting an epithelial origin for these tumors (Fig. 2). Consistent with our results, ovarian tumors formed in APC-PTEN knockout model were also negative for inhibin-α staining [6]. Recently, Fan *et al.* specifically activated β-catenin in granulosa cells of the mouse ovary by conditional deleting exon3 (Ex3) of β-catenin using Cyp19-cre and observed no tumor formation in these mutant mice (*Cyp19-Cre;Ctnnb1^Δ(ex3)/+^*) [53]. Furthermore, only membranous but not nuclear β-catenin expression is observed in human granulosa cell tumor patient samples [28], suggesting Wnt/β-catenin signaling does not play a role in either mouse or human granulosa cell tumor development. Alternatively, granulosa cells are thought to originate from the coelomic epithelium of the ovary during the perinatal period [54], which could classify the cells in these reports as “pregranulosa” cells that can differentiate into either granulosa cell tumors or ovarian mesenchymal tumors, depending on the context.

Lastly, our findings, along with those of others [55], provide an exciting prospect for using mTOR inhibitors together with other therapies in the treatment of ovarian carcinomas. mTOR is a critical regulator of cell growth and proliferation, and activation of this pathway occurs in many tumors including colon, ovarian, and uterine [19], [38], [41], [55]. Activation of Wnt/β-catenin has been shown to upregulate mTOR activity in colon and uterine cancers [19], [38], and treatment with an mTOR-specific inhibitor suppresses polyp formation in a colon cancer mouse model [38]. In this study, we have shown that activation of β-catenin and/or AKT increases mTOR activity and that inhibition of that activity with rapamycin suppresses the tumor burden by controlling cell proliferation and death. Everolimus (a rapamycin derivative) treatment has been shown to reduce tumor growth of cisplastin-resistant clear cell ovarian carcinoma cells [56] and both the onset and progression of ovarian cancer in a mouse model expressing SV40 Large T antigen driven by the MIS type II receptor (*Amhr2*) promoter [2], [56]. Presently, mTOR inhibitors are in phase I–III trials for other solid tumors [41]. In light of our studies, their use in OEA-specific trials should also be considered.

## Supporting Information

Figure S1 No abnormalities were present in the control (*Ctnnb1^Δ(ex3)/+^*) adult ovaries (A & B). In adult mutant (*Amhr2-Cre;Ctnnb1^Δ(ex3)/+^*) ovaries, cancerous lesions were present throughout the ovary and in the intrabursal space (arrowheads) (C–E). β-catenin staining in adult mutant ovaries (F). Bars represent 50 um.
(TIF)

Figure S2 H&E staining of 10-day old ovaries from control and mutant (*Amhr2-Cre;Ctnnb1^Δ(ex3)/+^;Pten^Δ/Δ^*) mice (Panel A–C). Representative section of tumor from 8 week-old *Amhr2-Cre;Ctnnb1^Δ(ex3)/+^;Pten^Δ/Δ^* mice (Panel D). Bars represent 50 um.
(TIF)

Figure S3 Rapamycin treatment of tumors decreased proliferation and increased cell death. Staining for pH3 (A–F, green), TUNEL (G–L, green), and activated caspase 3 (M–R, red) was performed on three different tumors derived from three different animals. Nuclei were counterstained with DAPI. Bars represent 50 um.
(TIF)
