# Supplementary figures and images for: Mammalian Target of Rapamycin Is a Therapeutic Target for Murine Ovarian Endometrioid Adenocarcinomas with Dysregulated Wnt/β-Catenin and PTEN
Source: PLoS One. 2011 Jun 9;6(6):e20715. doi: 10.1371/journal.pone.0020715 (PMC3111436; doi:10.1371/journal.pone.0020715)

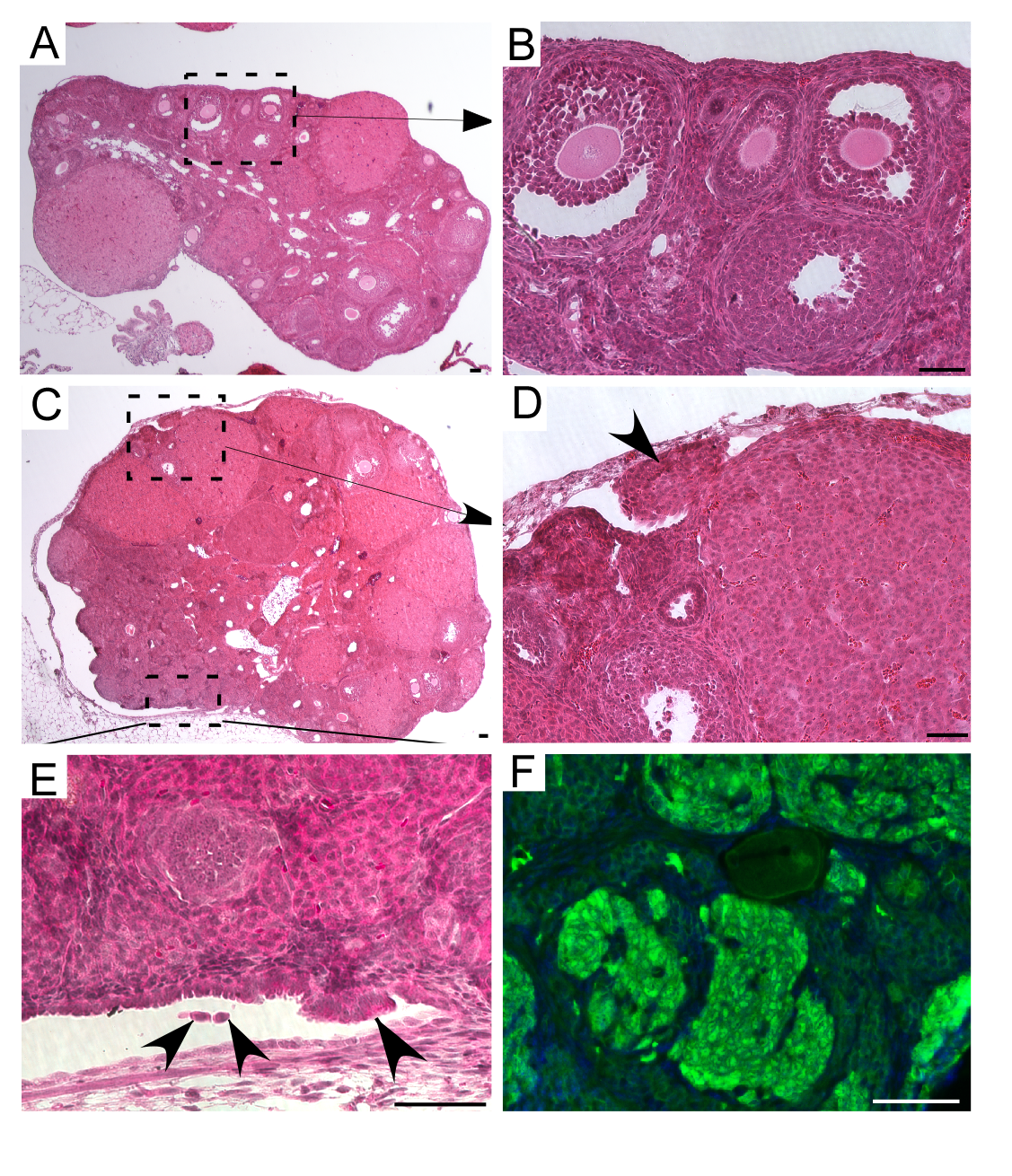

Supplement: Figure S1 — No abnormalities were present in the control (Ctnnb1Δ(ex3)/+) adult ovaries (A & B). In adult mutant (Amhr2-Cre;Ctnnb1Δ(ex3)/+) ovaries, cancerous lesions were present throughout the ovary and in the intrabursal space (arrowheads) (C–E). β-catenin staining in adult mutant ovaries (F). Bars represent 50 um. (TIF) [file pone.0020715.s001.tif]

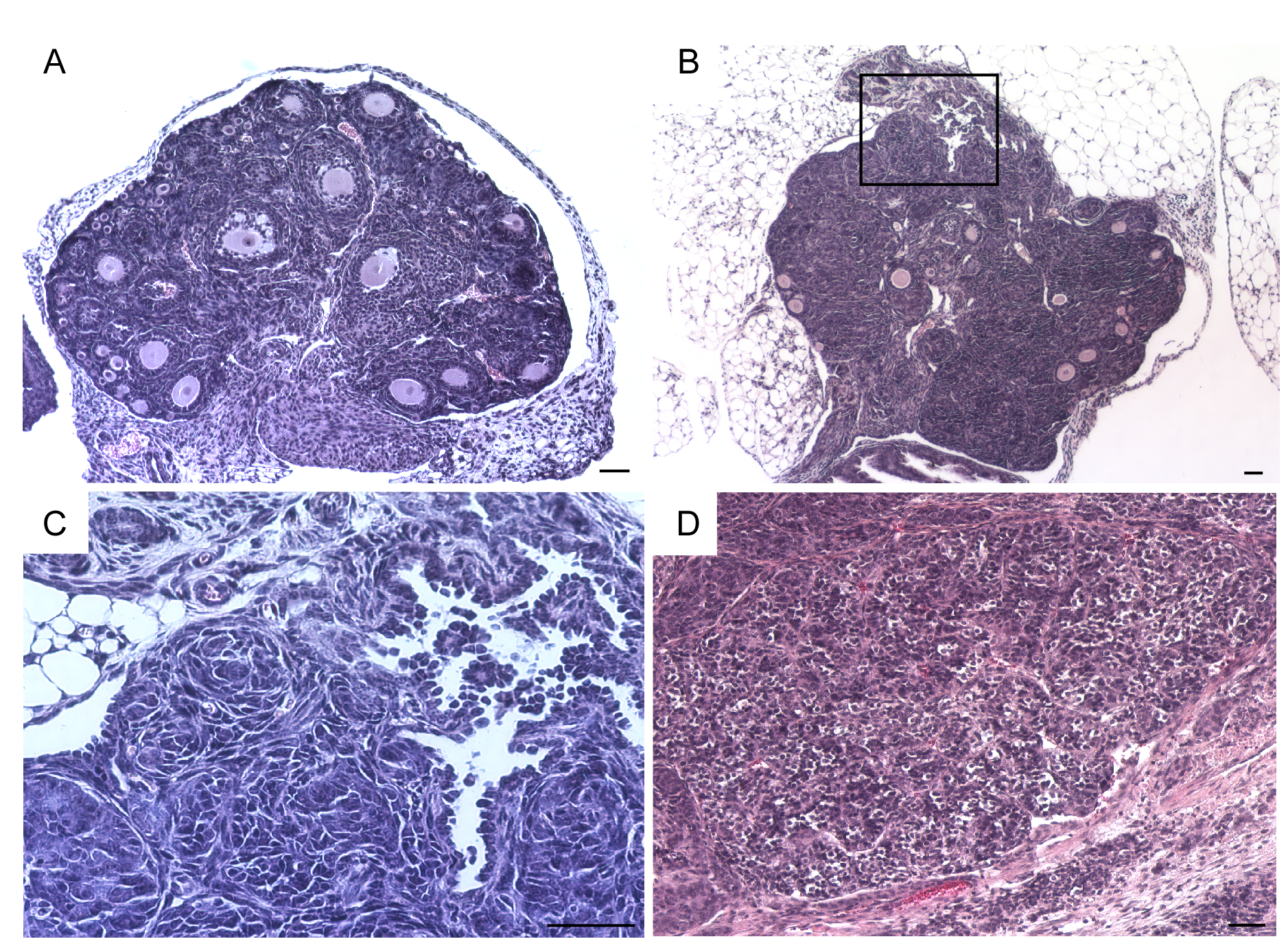

Supplement: Figure S2 — H&E staining of 10-day old ovaries from control and mutant (Amhr2-Cre;Ctnnb1Δ(ex3)/+;PtenΔ/Δ) mice (Panel A–C). Representative section of tumor from 8 week-old Amhr2-Cre;Ctnnb1Δ(ex3)/+;PtenΔ/Δ mice (Panel D). Bars represent 50 um. (TIF) [file pone.0020715.s002.tif]

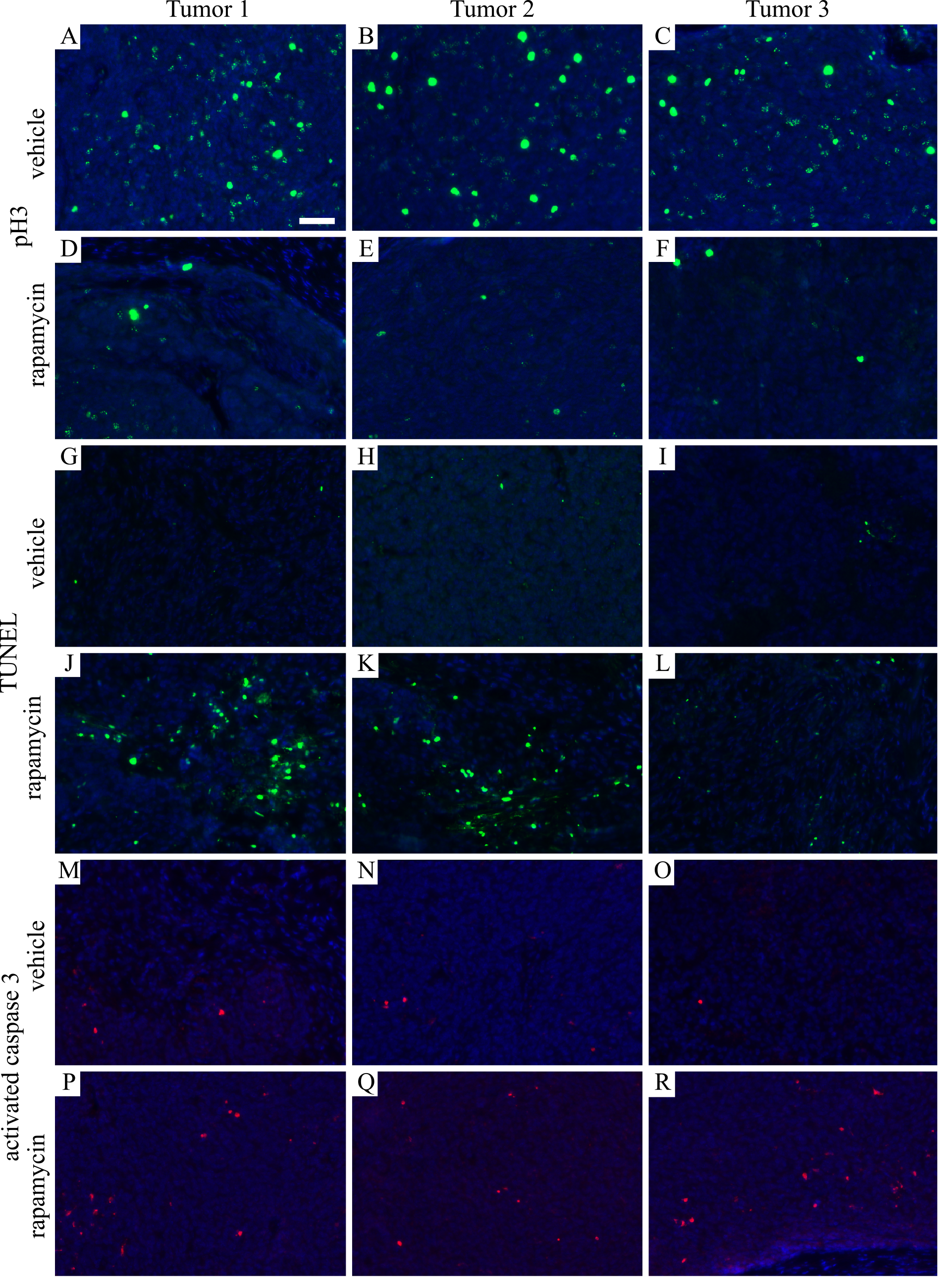

Supplement: Figure S3 — Rapamycin treatment of tumors decreased proliferation and increased cell death. Staining for pH3 (A–F, green), TUNEL (G–L, green), and activated caspase 3 (M–R, red) was performed on three different tumors derived from three different animals. Nuclei were counterstained with DAPI. Bars represent 50 um. (TIF) [file pone.0020715.s003.tif]
